# Maintenance of HDACs and H3K9me3 Prevents Arterial Flow-Induced Venous Endothelial Damage

**DOI:** 10.3389/fcell.2021.642150

**Published:** 2021-04-09

**Authors:** Ting-Yun Wang, Ming-Min Chang, Yi-Shuan Julie Li, Tzu-Chieh Huang, Shu Chien, Chia-Ching Wu

**Affiliations:** ^1^Department of Cell Biology and Anatomy, College of Medicine, National Cheng Kung University, Tainan, Taiwan; ^2^Department of Bioengineering, University of California, San Diego, La Jolla, CA, United States; ^3^Institute of Engineering in Medicine, University of California, San Diego, La Jolla, CA, United States; ^4^Institute of Basic Medical Sciences, College of Medicine, National Cheng Kung University, Tainan, Taiwan; ^5^International Center for Wound Repair and Regeneration, National Cheng Kung University, Tainan, Taiwan; ^6^Department of Biomedical Engineering, National Cheng Kung University, Tainan, Taiwan

**Keywords:** shear stress, venous endothelial cell, HDACs, H3K9me3, FAK, ITSA-1, EGF, vein graft failure

## Abstract

The transition of flow microenvironments from veins to arteries in vein graft surgery induces “peel-off” of venous endothelial cells (vECs) and results in restenosis. Recently, arterial laminar shear stress (ALS) and oscillatory shear stress (OS) have been shown to affect the cell cycle and inflammation through epigenetic controls such as histone deacetylation by histone deacetylases (HDACs) and trimethylation on lysine 9 of histone 3 (H3K9me3) in arterial ECs. However, the roles of H3K9me3 and HDAC in vEC damage under ALS are not known. We hypothesized that the different responses of HDACs and H3K9me3 might cause vEC damage under the transition of venous flow to arterial flow. We found that arterial ECs showed high expression of H3K9me3 protein and were retained in the G0 phase of the cell cycle after being subjected to ALS. vECs became round under ALS with a decrease in the expression of H3K9me3, HDAC3, and HDAC5, and an increase in the expression of vascular cell adhesion molecule 1 (VCAM-1). Inhibition of HDACs activity by a specific inhibitor, phenylbutyrate, in arterial ECs caused similar ALS-induced inflammation and cell loss as observed in vECs. Activation of HDACs and H3K9me3 by ITSA-1, an HDAC activator, could prevent ALS-induced peel-off and reduced VCAM-1 expression in vECs. Moreover, shear stress modulates EC morphology by the regulation of focal adhesion kinase (FAK) expression. ITSA-1 or EGF could increase phosphorylated (p)-FAK expression in vECs under ALS. We found that perturbation of the activity of p-FAK and increase in p-FAK expression restored ALS-induced H3K9me3 expression in vECs. Hence, the abnormal mechanoresponses of H3K9me3 and HDAC in vECs after being subjected to ALS could be reversed by ITSA-1 or EGF treatment: this offers a strategy to prevent vein graft failure.

## Introduction

Autologous saphenous veins are the most commonly used conduits in coronary artery bypass grafts for the treatment of coronary artery stenosis (De Vries et al., 2016; McKavanagh et al., 2017; Caliskan et al., 2020). Unfortunately, low efficacy of grafting can occur due to vascular inflammation, thrombosis, intimal hyperplasia, and subsequent accelerated atherosclerosis, *viz.* “vein graft failure” (VGF) or “vein graft disease” (VGD) (De Vries et al., 2016; de Vries and Quax, 2018; Ruiter and Pesce, 2018). Moreover, failure of grafts increases the frequency of adverse cardiovascular outcomes and death. Another type of VGF can be seen in arteriovenous grafts for hemodialysis. A radiocephalic arteriovenous fistula (AVF) at the wrist is the first choice for hemodialysis access. In radiocephalic AVFs, the A-V angle between the vein and the proximal artery of juxta-anastomotic region wider than 46.5° leads to disturbed blood flow, which is the most common site of venous stenosis (Yang et al., 2020).

Endothelial cells (ECs) line the inner wall of vessels. They have a crucial role in the maintenance of vessel structure and homeostasis (Shimokawa and Satoh, 2014; Peng et al., 2019). ECs can sense mechanical forces (“mechanosensing”) and convert them into intracellular signals for cell remodeling by transmitting the signal across the transmembrane adhesion receptors (e.g., cadherin, syndecan, and integrin) (Chien, 2007; Garoffolo and Pesce, 2019; Peng et al., 2019). Therefore, maintenance of a physiologic, laminar shear stress is crucial for normal vascular function, including regulation of vascular caliber as well as inhibition of cell proliferation, thrombosis, and inflammation of vessel walls (Frosen et al., 2019). Venous endothelial cells (vECs) are physiologically adapted to chronic low shear stress. When vECs are implanted into the arterial circulation, they are exposed suddenly to arterial laminar shear stress (ALS). ALS can induce the molecular cascades of autophagy and apoptosis in vECs to cause endothelial dysfunction and stenosis recurrence (Chang et al., 2016). Endothelial dysfunction is associated with cardiovascular risk factors and can initiate atherosclerosis (Hadi et al., 2005; Garoffolo and Pesce, 2019). Several factors can cause endothelial dysfunction. Nitric oxide acts as a vasodilator, and it has been reported that reduced expression of nitric oxide damages EC function (Gimbrone and Garcia-Cardena, 2016; Tajadura et al., 2020). Loss of anchorage also results in endothelial dysfunction. Integrin receptors in focal adhesions (FA) mediate cell adhesion and contribute to the integrity of the endothelial barrier. Laminar shear stress induces FA remodeling by forming peripheral “actin bundles” on the basal side of the cell and subsequently causes cell disassociation. Disruption of FA signaling leads to loss of cell adhesion and triggers cell death (Kirchenbuechler et al., 2014; Verma et al., 2015; Driscoll et al., 2020).

Several studies have shown that shear stress can regulate homeostasis, proliferation, apoptosis, migration, and remodeling in ECs, as well as gene expression (Li et al., 2005; Peng et al., 2019). Proper laminar shear stress stimulates cellular responses (e.g., secretion of cytokines and growth factors) to promote antithrombosis and antigrowth, and further maintain cell function. However, a sudden change in shear stress can cause EC apoptosis by increasing secretion of proinflammatory factors [e.g., monocyte chemoattractant protein 1 and vascular cell adhesion molecule 1 (VCAM-1)] and is associated with several pathophysiological conditions (e.g., atherosclerosis). Cell cycle is an ordered set of events that leads to duplication of a cell’s DNA and cell division to produce two daughter cells. The cell cycle comprises four phases: two gap phases (G1 and G2), an S phase (for DNA synthesis), and an M phase (which consists of genetic material), followed by cell division. There is increasing evidence for the importance of cell cycle dysregulation in the pathogenesis of cancer, atherosclerosis, inflammation, and neurodegenerative disorders (Zhivotovsky and Orrenius, 2010; Peng et al., 2019). Several investigations have indicated that shear stress inhibits DNA synthesis in ECs by inhibiting transition from the G0/G1 to S phase (Akimoto et al., 2000). However, it has been reported that disturbed flow promotes cell cycle progression and maintains cells in the S + G2/M phase (Guo et al., 2007). Therefore, derangement of steady shear stress leads to cell proliferation, which may cause atherosclerosis through disruption of EC stability (Akimoto et al., 2000). The stages of the cell cycle are regulated by epigenetic modification (Dominguez and Berger, 2008), but the mechanism of assembly and dynamics of histone modification in cell cycle regulation are not known.

Post-translational modification (PTM) includes DNA methylation, histone modifications, and RNA interference. PTM can also modulate the actin cytoskeleton to affect cell functions (e.g., cell adhesion) (Yan et al., 2010; Kottakis et al., 2011). Recent studies have shown that KDM2B (a histone demethylase for H3K4me3 and H3K36me2) regulates the expression of focal adhesion kinase (FAK), phosphoinositide 3 kinase (PI3K), and protein kinase B (Akt) (Zacharopoulou et al., 2018). Luo et al. (2011) have shown that interaction between FAK and methyl CpG-binding protein 2 (MeCP2) can modify heterochromatin reorganization and suppress the association between MeCP2 and histone deacetylase 1 (HDAC1) during oxidative stress. FA dynamics are important for survival and spread of ECs and are associated with actin organization (Carragher and Frame, 2004). Therefore, disruption of FA signaling may lead to loss of cell adhesion and trigger cell death (Caltagarone et al., 2007). FA, a macromolecular complex comprising talin, α-actinin, vinculin, zyxin, paxillin, Src, and FAK, is the connection between the extracellular matrix and actin cytoskeleton (Kuo, 2013). FA mediates the adhesion, migration, mechanosensing, and signaling of cells (Case et al., 2015). FAK is a cytoplasmic tyrosine kinase that mediates the dynamics and signaling of FA in response to growth factors and integrin-ligand binding (Li et al., 2002). Phosphorylation of FAK at Tyr-397 is important for recruitment of Src to promote the formation and maturation of FA. Also, FAK drives FA turnover through control of targeted proteolysis of FA proteins (Romer et al., 2006). FAK is required to maintain EC function, and knockdown of FAK in ECs increases the apoptosis and permeability of cells (Zhao et al., 2010). Therefore, the different responses of FAK between arterial and venous ECs under ALS may be one of the reasons for EC damage. However, the mechano-transduction pathway between arterial and venous ECs under ALS is not known.

Here, we compared the differences in HDACs and trimethylation of lysine 9 on histone 3 (H3K9me3) in arterial ECs and venous ECs under ALS. We speculated that FAs-mediated epigenetic regulation may also cause the vEC damage under ALS.

## Materials and Methods

### Cell Lines

The human umbilical vein endothelial cells (HUVECs) (BCRC no. H-UV001) and the human saphenous vein endothelial cells (HSVECs) (cat. no. C-12231) were purchased from Bioresource Collection and Research Center (Hsinchu, Taiwan) and PromoCell (Heidelberg, Germany), respectively. All cells were cultured in Medium 199 (M199) containing 20% fetal bovine serum (FBS), 100 U/ml penicillin, 100 μg/ml streptomycin (P/S), and 25% endothelial cell growth medium (EGM) in an atmosphere of 5% CO_2_ at 37°C. When cells reached a 90% confluent monolayer, cell passaging was performed by trypsinization using 0.05% trypsin-EDTA. The cells used in experiments were from passages 3 to 7.

### Shear Stress *in vitro*

The shear stress experiments were conducted based on our previous study (Wu et al., 2007). Cells were seeded on fibronectin (10 μg/ml)-coated slides for 24 h and then starved in M199 medium containing 2% FBS. After 24 h, the starved ECs were transferred to M199 medium containing 20% FBS and 25% EGM and maintained under static (ST) condition or subjected to ALS or oscillatory shear stress (OS). For the ALS or OS group, the slides with ECs were assembled into the flow chamber, in which a flow channel was created by “sandwiching” a silicone gasket between the slide and an acrylic plate. The CO_2_-equilibrated medium flew across the flow chamber in response to the pressure difference between the inlet and outlet of the chamber. Cells were subjected to ALS at 12 dynes/cm2 or OS at 0.5 ± 4 dynes/cm2 for 12 h or 24 h. The total area subjected to shearing was 1.5 cm in width (W) and 5.0 cm in length (L). The channel height (h) was 0.025 cm, and the shear flow in this narrow gap was laminar with a parabolic velocity. The wall shear stress (τ wall) was calculated as: τ wall = ΔP (h/2L) = 6Qμ/Wh^2^, where ΔP is the pressure difference between the inlet and the outlet of the flow channel, Q is the volumetric flow rate, and μ is the fluid viscosity. Protein samples were collected for Western blotting analysis.

### Western Blot Analysis

The ECs were washed twice with ice-cold phosphate-buffered saline and then lysed by using ice-cold lysis buffer containing Tris (50 mM, pH 7.4), NaCl (150 mM), and a protease inhibitor cocktail. The protein concentration was determined using a protein assay kit based on the Bradford method. Total protein (25 μg) in cell lysates was separated by sodium dodecyl sulfate–polyacrylamide gel electrophoresis using 10% gels and transferred to nitrocellulose membranes. The latter were blocked by 5% skimmed milk Tris-buffered saline with Tween 20 (TBST) for 2 h at room temperature and then immunoblotted with primary specific antibodies overnight at 4°C. The primary antibodies are as follows: H3K9me3 (1:1000, Abcam), cyclin A1 (1:1000, Cell Signaling), cyclin B1 (1:1000, Cell Signaling), HDAC1 (1:1000, Abcam), HDAC2 (1:1000, Abcam), HDAC3 (1:500, Santa Cruz), HDAC4 (1:500, Santa Cruz), HDAC5 (1:500, Santa Cruz), phosphor-HDAC3 (p-HDAC3, 1:1000, Cell Signaling), phosphor-HDAC4,5,7 (p-HDAC4,5,7, 1:1000, Cell Signaling), VCAM-1 (1:1000, Santa Cruz), phosphor-FAK Y397 (p-FAK, 1:1000, BD Biosciences), and total FAK (t-FAK, 1:1000, BD Biosciences). Thereafter, the membrane was hybridized with secondary antibodies conjugated with horseradish peroxidase (HRP) (Sigma-Aldrich). Signals were developed with the ECL Ultra Western HRP Substrate (Immobilon^TM^; Merck, Whitehouse Station, NJ, United States) and X-ray films (Fujifilm; Tokyo, Japan).

### Transfection by Electroporation

A total of 1 × 10^6^ ECs were suspended in electroporation buffer (0.1 ml of Opti-MEM, Gibco) and mixed with plasmids (5 μg). This mixture of ECs and plasmid was added into 2-mm gap cuvettes. Electroporation was carried out with a poring pulse of 175 V for a pulse length of 5 ms and a transfer pulse of 20 V for 50 ms. After electroporation, the cells were seeded in a 6-mm fibronectin-coated slide with 0.5 ml of M199 medium supplemented with 20% FBS without antibiotics. After 24 h of serum starvation, the slides were assembled in the flow chamber and subjected to fluid shear.

### Fluorescence Resonance Energy Transfer

Fluorescence Resonance Energy Transfer (FRET) is a distance-dependent physical process in which energy is transferred from one fluorophore (donor) to another (acceptor). Donor leakage is determined from cyan fluorescent protein (CFP)-transfected cells. Cross-excitation of acceptors is obtained from yellow fluorescent protein (YFP)-transfected cells. FRET is dependent on the distance and relative orientation between the two fluorophores (CFP and YFP) (Wang and Wang, 2009). FRET maps and the pixel-wise FRET Index for the sensors were determined using the FRET ratio (= FRET channel/CFP channel).

To monitor the dynamics of H3K9 trimethylation, a FRET-based H3K9me3 biosensor kindly provided by Dr. YingXiao Wang (University of California, San Diego, La Jolla, United States) was used (Lin et al., 2004). The FRET-based H3K9me3 biosensor comprised a YFP tag, HP1, CFP tag, and H3 N-terminus. When the H3K9me3 biosensor was activated, the YFP-tagged HP1 interacted with the K9 methylation from the CFP-tagged H3 N-terminus. This action led to the proximation of CFP to YFP, resulting in an increase in the FRET signal. In contrast, the FRET ratio was reduced by the removal of YFP-HP1 from CFP-H3K9 when H3K9me3 was deactivated. The time-lapse images were collected by confocal microscopy with a 44DF20 excitation filter, a 455DRLP dichroic mirror, and two emission filters controlled by a filter changer (480DF30 for CFP and 535DF25 for YFP) with a time interval of 1 h. To achieve high-quality fluorescence images of living cells, a 60 × oil objective lens was used in a confocal microscope with environmental control for supplementation with 5% CO_2_ at 37°C. Images were analyzed by FluoCell (Liu et al., 2014), which has been developed by the research team of Dr. YingXiao Wang. The FRET ratio was calculated based on the FRET/CFP signal using MATLAB software^1^.

### Phalloidin F-actin Staining

Arterial laminar shear stress-treated vECs were washed with PBS and fixed in 4% paraformaldehyde in PBS at room temperature for 10 min. Then, the fixation solution was aspirated and the cells were washed twice. Alexa fluor 546-Phalloidin working solution was added for 60 min. Cells were washed with PBS and fixed by using mounting media with DAPI. Samples were examined using the Olympus FluoView FV1000 confocal microscope (Olympus, Tokyo, Japan). Images were analyzed using the Olympus FluoView FV10-ASW software (Olympus).

### Statistical Analysis

Statistical analysis was carried out using Prism 6 software (GraphPad, La Jolla, CA, United States). *p* < 0.05 was considered to be statistically significant in this study. The specific statistical methods are indicated in each figure legend.

## Results

### Flow-Modulated H3K9me3 Expression Plays an Important Role in Cell Cycle Regulation

**FIGURE 1:**
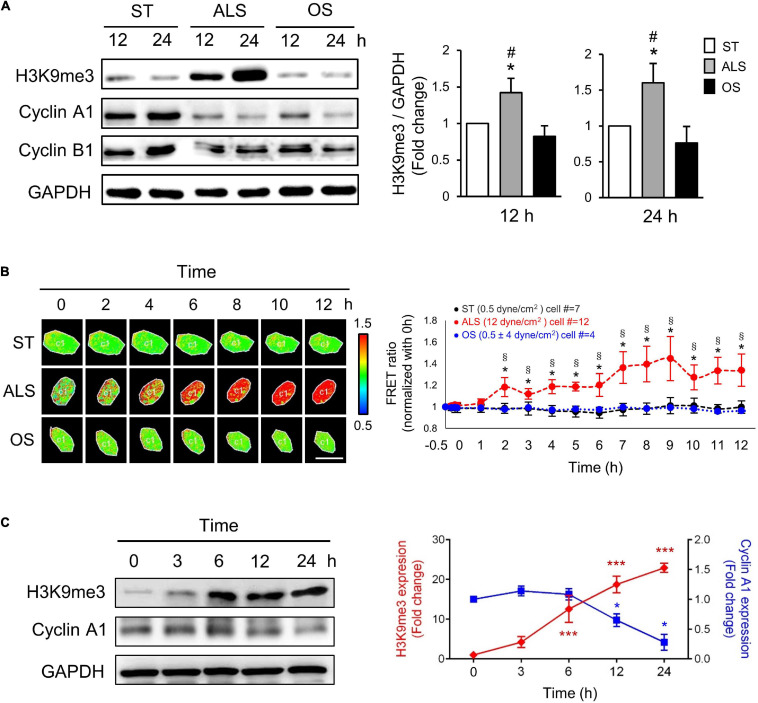
ALS increased the expression of H3K9me3 and promoted the cell quiescence in arterial ECs. **(A)** Western blotting analysis for the expression of H3K9me3, cyclin A1, and cyclin B1 in HUVECs, which were subjected to ST, ALS, or OS treatment for 12 and 24 h. ALS increased the H3K9me3 expression and decreased the cyclin A1 expression, whereas OS did not change the H3K9me3 at 12 and 24 h. **(B)** The time-lapse FRET ratio images of arterial ECs expressing the H3K9me3 biosensors were recorded to track the status of H3K9me3 every 2 h under ST, ALS, or OS over the time course of 12 h. The results were normalized to 0 h for each experiment. Scale bar in the phase image: 20 μm. **(C)** Western blotting analysis for the expression of H3K9me3 and cyclin A1 in synchronized HUVECs at 0, 3, 6, 12, and 24 h under ALS stimulation. The low expression of cyclin A1 was accompanied by the high level of H3K9me3. Data were represented as mean ± SEM, *n* = 3. *p*-values were calculated using one-way ANOVA in **(A)** and **(C)** and using two-way ANOVA in **(B)** with Tukey’s multiple comparisons post-tests. In **(A)** and **(B)**, *p < 0.05, ALS-treated HUVECs compared to the ST group; ^#^*p* < 0.05, ALS-treated HUVECs compared to the OS group. **(C)** *p < 0.05, ***p < 0.001 with different color indicates a statistically significant difference at each time point compared to their corresponding control group (0 h).

Several reports have revealed that ALS plays a critical role in inhibiting the cell cycle event from the G0/G1 phase to the S phase, which maintains cell quiescence (Ehsan et al., 2002; Luo et al., 2011). To investigate the role of H3K9me3 in the ALS-induced cell quiescence, first, we examined the effect of different shear stresses in modulating H3K9me3 expression in ECs. The HUVECs, which are more like arterial ECs, were cultured under ST (no flow), ALS, or OS conditions for 12 or 24 h; the expressions of cyclin A1 (which is involved in the control of the transition from G1 to S phase and mitosis), cyclin B1 (which is required for entry into and progression through mitosis), and H3K9me3 were measured by Western blotting (Figure 1A). ALS increased H3K9me3 expression and inhibited cyclin A1 expression in HUVECs, indicating that ECs could not enter DNA synthesis and were retained in the G0 phase. In contrast, OS did not alter H3K9me3 expression and caused HUVEC proliferation.

To explore the real-time status of H3K9me3 in arterial ECs under different conditions, we transfected the CFP-YPet H3K9me3 FRET biosensor into HUVECs and utilized the time-lapse confocal microscopy to monitor H3K9me3 in HUVECs under different conditions. In HUVECs subjected to ALS, time-lapse images showed that the FRET density of H3K9me3 biosensor in nuclei increased gradually from 2 to 12 h after (Figure 1B). However, there was no change in the FRET ratio for H3K9me3 in ST- or OS-treated HUVECs (Figure 1B). The quantified mean ratio for normalized FRET intensity showed a significant increase under ALS in comparison with that under ST or OS. These results suggested that H3K9me3 expression was induced by ALS, but not ST or OS, in arterial ECs. We also demonstrated correlation between cell cycle and H3K9me3 in arterial ECs by synchronization under ST (Figure 1C). After 24 h of serum starvation, HUVECs were cultured in normal medium, and protein was collected at 0, 3, 6, 12, and 24 h under ALS stimulation. The expression of H3K9me3 in HUVECs was negatively correlated with that of cyclin A. Taken together, these data suggested that ALS could induce H3K9me3 expression and quiescence in arterial ECs.

### ALS Induced Distinct Effects in Arterial and Venous ECs

**FIGURE 2:**
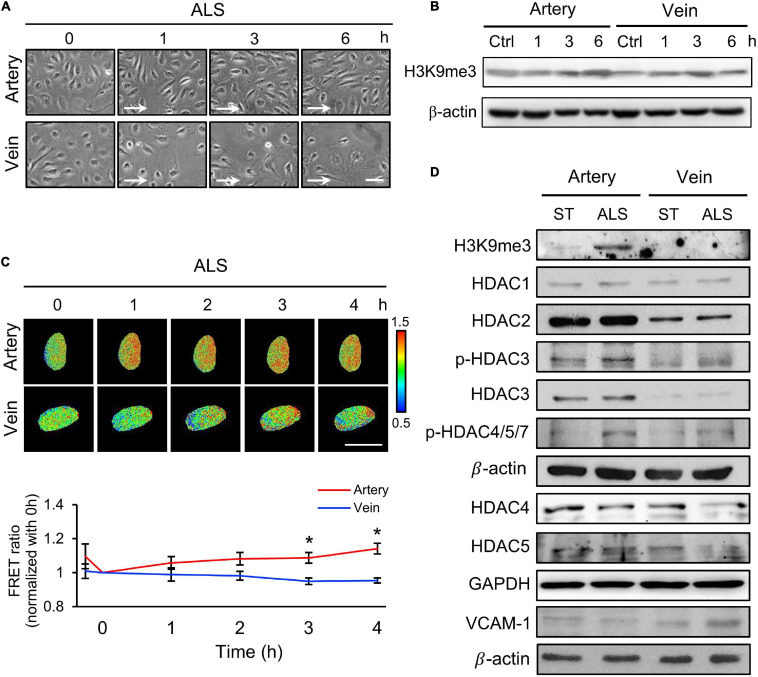
The different effect of arterial and venous ECs under ALS. **(A)** Morphology observations of arterial ECs (HUVECs) and venous ECs (HSVECs) after being subjected to ALS for 0, 1, 3, and 6 h. **(B)** The Western blot analysis for the expression of H3K9me3 and β-actin in total cell lysates from **(A)**. The expression of H3K9me3 was time-dependent increase in arterial ECs but not in vECs. **(C)** The time-lapse FRET ratio images of HUVECs and HSVECs expressing the H3K9me3 biosensors were recorded to track the status of H3K9me3 every 2 h under ALS for 12 h. Compared to HUVECs, no FRET ratio of activated H3K9me3 was observed in vECs upon ALS stimulation at different time points (0, 1, 3, and 6 h). **(D)** Protein expressions were examined by using Western blotting analysis. HUVECs and HSVECs were subjected to ST or ALS condition for 24 h. Lower expression of H3K9me3 in ALS-treated HSVECs compared to ALS-treated HUVECs was shown. ALS decreased the HDAC4 and HDAC5 and increased VCAM-1 in ALS-treated HSVECs. The increases of HDAC3, HDAC4, HDAC5, p-HDAC3, and p-HDAC4,5,7 were observed in ALS-treated HUVSCs at 24 h. Scale bar in **(A)** = 200 μm. Arrow indicated the flow direction of ALS (left to right). Scale bar in **(C)** = 20 μm. Data were represented as mean ± SEM. *p-values were calculated using one-way ANOVA with Tukey’s multiple comparisons post-tests. *p < 0.05 compared to the vECs group.

Next, we examined the relation between the expression of H3K9me3 under ALS and inflammatory response in vECs, with the aim of comparing the responses of arterial and venous ECs under ALS. HUVECs and HSVECs were subjected to ALS for 0, 1, 3, and 6 h. Phase images showed that ALS triggered arterial ECs to become elongated and align parallel to the flow direction (Figure 2A). In contrast, vECs became rounded and were lost upon ALS stimulation. Western blotting results showed that ALS resulted in sustained increases in H3K9me3 expression in arterial ECs, but not in vECs (Figure 2B). These results demonstrated that H3K9me3 expression in response to ALS was different between arterial and venous ECs. To confirm this finding, we transfected the H3K9 FRET biosensor into HUVECs or HSVECs to explore the status of H3K9me3. Time-lapse images were recorded 0, 1, 2, 3, and 4 h after ALS. The H3K9me3 FRET ratio of HUVECs increased in response to ALS stimulation. In contrast, the H3k9me3 FRET ratio decreased after ALS in HSVECs (Figure 2C).

Several studies have demonstrated that HDACs have crucial roles in EC inflammation and atherosclerosis (Zhou et al., 2011; Lee and Chiu, 2019). Hence, we examined the expressions of HDAC1, HDAC2, HDAC3, HDAC4, and HDAC5 and an inflammation marker (VCAM-1) in these two cell types following ALS treatment. ALS reduced the expressions of HDAC4 and HDAC5 in HSVECs, while it increased the expressions of HDAC3, HDAC4, HDAC5, phosphorylated (p)-HDAC3, and p-HDAC4/5/7 in HUVECs (Figure 2D). The expression of VCAM-1 in HSVECs after ALS was higher than that in HUVECs. Taken together, these results indicate that ALS induced different effects in arterial and venous ECs. In addition, the results suggest the involvement of H3K9me3 and HDAC in the ALS-induced inflammatory response in vECs.

### Inhibition of HDAC Activity Reversed an Atheroprotective Morphology and Induced Inflammation in ALS-Treated Arterial ECs

**FIGURE 3:**
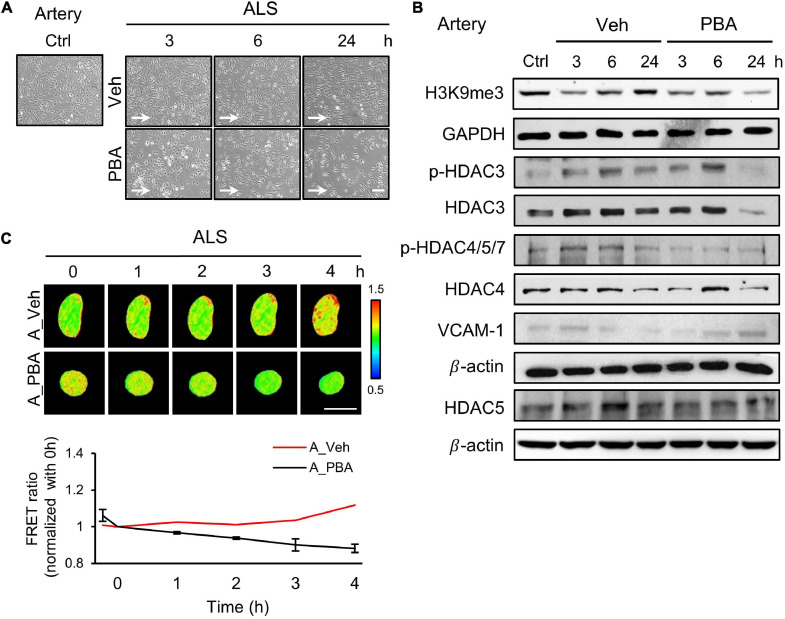
PBA inhibited the expression of H3K9me3 in arterial ECs. **(A)** The images were presented the cell morphology of PBA (150 μM) or vehicle-treated arterial ECs (HUVECs) under ALS at 3, 6, and 24 h. PBA-induced arterial ECs had gradually increased the number of round-up damage morphology after subjected to ALS at 3, 6, and 24 h. **(B)** Protein expressions were examined by using Western blotting analysis. The decreases of H3K9me3, p-HDAC3, p-HDAC4,5,7, HDAC3, HDAC4, and HDAC5 and the increase of VCAM-1 were observed after treating PBA under ALS in HUVECs compared to vehicle group. **(C)** By utilizing the H3K9me3 FRET biosensor to examine the H3K9me3 status, the FRET ratio of H3K9me3 was declined in PBA-treated HUVECs under ALS condition compared to the vehicle group **(C)**. Scale bar in **(A)** = 200 μm. Scale bar in **(C)** = 20 μm. Data were represented as mean ± SEM. Arrow indicated the flow direction of ALS (left to right).

To further assess the role of HDACs in the responses of arterial ECs under ALS, we perturbed HDACs expression by treating HUVECs with a HDAC inhibitor, sodium phenylbutyrate (PBA), and subjected PBA-treated HUVECs to ALS for 0, 1, 3, and 6 h. Many PBA-treated HUVECs were lost as comparison with the vehicle group (Figure 3A). Western blot results revealed that the phosphorylation of HDAC3 and HDAC4/5/7 and the expression of HADC3, HDAC4, and HDAC5 were decreased by PBA treatment under ALS (Figure 3B). H3K9me3 expression was not increased in PBA-treated HUVECs after being subjected to ALS. An increase in VCAM-1 expression suggests that the abolishment of HDACs and H3K9me3 by PBA treatment caused inflammation in HUVECs under ALS. Using the H3K9 FRET biosensor, the status of H3K9me3 was investigated in PBA-treated HUVECs under ALS. The H3K9me3 FRET biosensor result showed a suppression of H3K9me3 expression in ALS-stimulated HUVECs upon PBA treatment (Figure 3C). These findings suggest that the inhibition of HDACs expression by PBA in arterial ECs suppressed the ALS-induced H3K9me3 expression and led to cell inflammation.

### Activation of HDACs Rescued vECs From the ALS-Induced Pathological Responses

**FIGURE 4:**
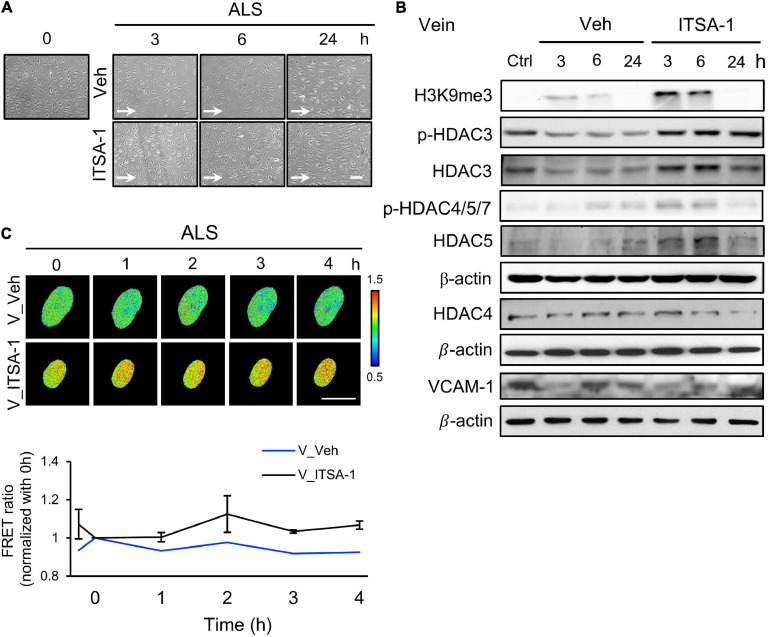
Reverse the ALS-induced inflammation by ITSA-1 in vEC. **(A)** The cell images of 150 μM ITSA-1- or vehicle-treated vECs (HSVECs) were shown under ALS for 0, 3, 6, and 24 h. The ITSA-1-treated HSVECs were significantly aligned parallel to the flow direction of ALS at 24 h. **(B)** Protein expression was analyzed by using Western blotting assay against H3K9me3, p-HDAC3, p-HDAC4,5,7, HDAC3, HDAC4, HDAC5, and VCAM-1. ITSA-1-treated HSVECs reduced the VCAM-1 expression by increasing the HDAC3, p-HDAC4, 5, 7, and H3K9me3 as compared to the vehicle group after exposed to ALS at 3 and 6 h. **(C)** The level of H3K9me3 was raised in the ITSA-1 group but not in vehicle group under ALS in H3K9me3 FRET biosensor-transfected HSVECs. Scale bar in **(A)** = 200 μm. Scale bar in **(C)** = 20 μm. Data were represented as mean ± SEM. Arrow indicated the flow direction of ALS (left to right).

Since we observed a decrease of HDACs expression in ALS-induced vEC inflammation (Figure 2D), we induced HDACs expression in HSVECs under ALS by initiating ITSA-1 (150 μM) treatment and measured H3K9me3 expression and cell inflammation in ISTA-1 and ALS treatments. Cell morphology was aligned parallel to the flow direction of ALS after ITSA-1 treatment in HSVECs (Figure 4A), indicating that ITSA-1 reverted the pathological responses to ALS in vECs. Western blotting showed that ITSA-1 increased the expressions of H3K9me3, pHDAC3, HDAC3, and p-HDAC4/5/7 in ALS-treated HSVECs (Figure 4B). A decrease in VCAM-1 expression was found after ITSA-1 treatment in HSVECs under ALS. These results suggested that the increases in expressions of HDACs and H3K9me3 through HDAC activation rescued vECs from ALS-induced inflammation. We used the H3K9me3 FRET biosensor to measure H3K9me3 expression in ITSA-1-treated HSVECs upon ALS stimulation. The normalized FRET ratio revealed that H3K9me3 expression was increased in the ITSA-1 group as compared with that in the vehicle group under ALS in HSVECs (Figure 4C). The FRET results are in accordance with the Western blotting results. Taken together, these data suggest that the increased expressions of HDACs and H3K9me3 by ITSA-1 could reverse inflammation in ALS-induced vECs.

### Effects of FAK on H3K9me3 Expression Under ALS

**FIGURE 5:**
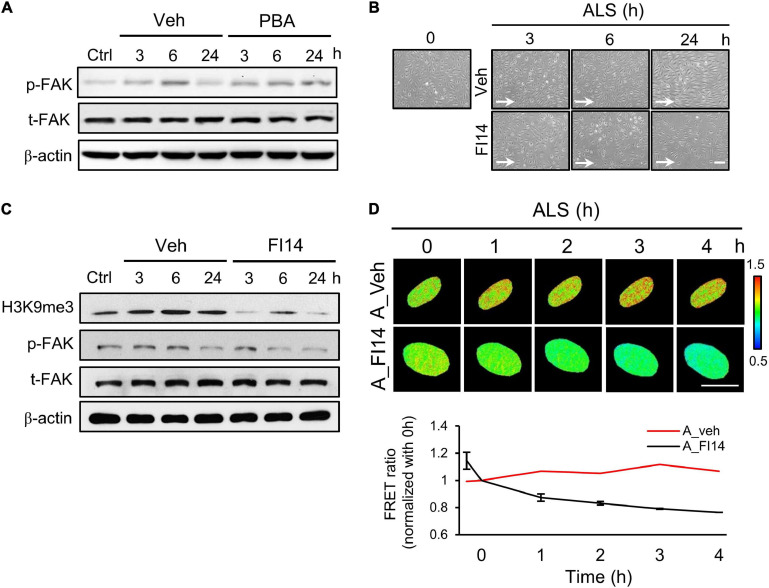
Inhibition of FAK decreased the ALS-induced H3K9 methylation in arterial ECs. **(A)** The expression of p-FAK was decreased in PBA (150 μM)-treated arterial ECs (HUVECs) by Western blotting assay after being subjected to ALS at 0, 3, 6, and 24 h. **(B)** ALS significantly triggered the cell peel-off in FI14-treated HUVECs at 24 h. **(C)** Protein expression was analyzed by using Western blotting assay against H3K9me3, p-FAK, and t-FAK. Blocking the phosphorylation of FAK by FI14 treatment decreased the expression of H3K9me3 in ALS-stimulated HUVECs. **(D)** The normalized H3K9me3 FRET ratio was declined in FI14-treated HUVECs after being subjected to ALS by transfecting the H3K9me3 FRET biosensor. Scale bar in **(B)** = 200 μm. Scale bar in **(D)** = 20 μm. Data were represented as mean ± SEM. Arrow indicated the flow direction of ALS (left to right).

It has been reported that ECs undergo cell death if they become detached from the extracellular matrix (Ruoslahti and Reed, 1994). Moreover, FAK is involved in the regulation of FA structures (Sieg et al., 1999) and the increase of integrin activation to strengthen FA (Michael et al., 2009). We explored whether p-FAK was involved in ALS-mediated H3K9me3 expression in arterial ECs. Western blotting showed that FAK phosphorylation in PBA-treated arterial ECs was decreased compared to the vehicle group under ALS (Figure 5A). We also examined the role of FAK in H3K9me3 expression under ALS by using a FAK inhibitor (FI14) and a FAK activator [epidermal growth factor (EGF)] in HUVECs and HSVECs. Arterial ECs became round and lost after using FI14 (5 μM) to inhibit the phosphorylation site (Y397) of FAK under ALS (Figure 5B). Cell images showed that FI14 treatment caused arterial ECs to peel off after exposure to ALS. Expressions of p-FAK, t-FAK, and H3K9me3 were measured by Western blotting (Figure 5C). A decrease of p-FAK was found upon FI14 treatment in HUVECs under ALS. H3K9me3 expression also declined after being subjected to ALS in FI14-treated HUVECs, indicating that the blockage of p-FAK expression could suppress ALS-induced H3K9me3 expression in arterial ECs. To confirm this result, we utilized the H3K9me3 FRET biosensor to detect the activation status of H3K9me3. The normalized FRET ratio was decreased after FI14 treatment in arterial ECs. During ALS stimulation, the results for the FRET biosensor of FI14-treated arterial ECs showed a similar trend to those for the Western blotting data (Figure 5D).

**FIGURE 6:**
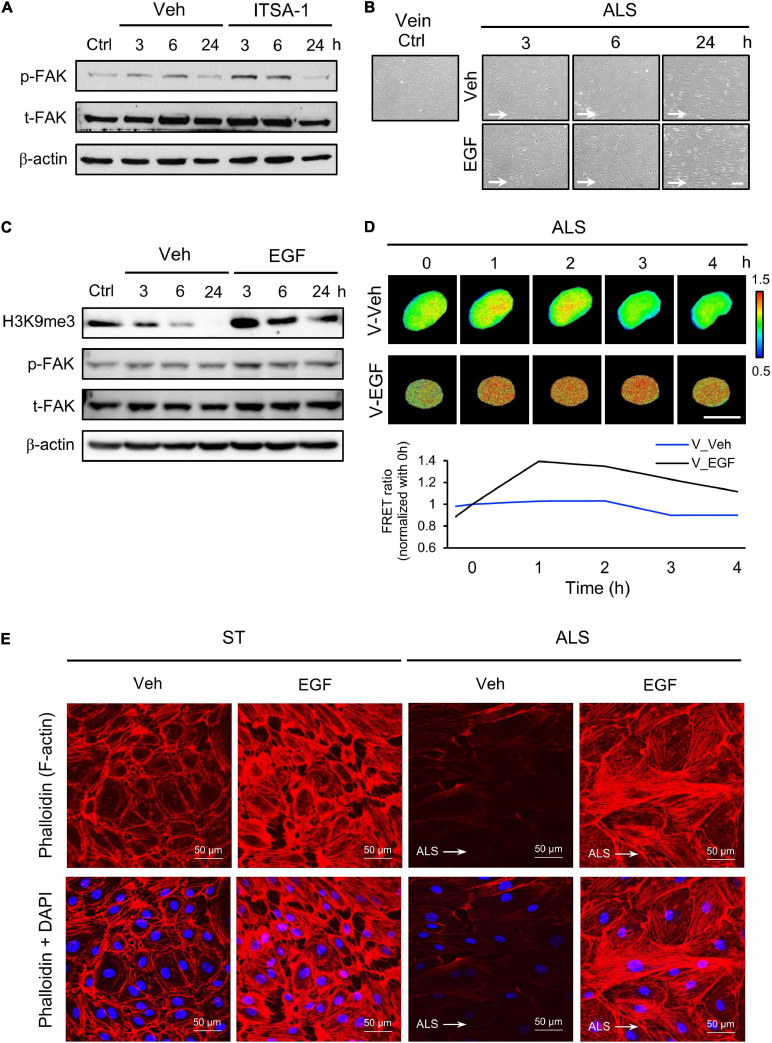
Protective effect of EGF on preventing ALS-induced EC inflammation. **(A)** Western blotting analysis for activating H3K9me3 by treating ITSA-1 in vEC (HSVECs); the expression of p-FAK was increased after ALS stimulation. **(B)** Treating EGF (100 ng/ml) in HSUVEs prevented the cell peel-off and the cells were parallel to the flow direction under ALS. **(C)** Protein expression was analyzed by using conventional Western blotting assay against H3K9me3, p-FAK, and t-FAK. The expression of H3K9me3 was gradually increased by raising the expression of p-FAK in EGF-treated HSVECs under ALS. **(D)** To validate the flow regulation of H3K9me3 in EGF-treated HUVECs, the results also confirmed that EGF induced H3K9me3 over the time course of 4 h in H3K9me3 biosensor-transfected HSVECs under ALS. **(E)** Alexa fluor 546-Phalloidin staining was used to determine the expression of F-actin on vECs with or without EGF treatment under ALS for 24 h. Nuclei were stained with DAPI (blue). Scale bar in cell phase images = 200 μm. Scale bar in FRET images = 20 μm. Data were represented as mean ± SEM. Arrow indicated the flow direction of ALS (left to right).

We also assessed the expression of p-FAK upon ITSA-1 treatment in ALS-stimulated HSVECs. FAK phosphorylation was increased by applying ITSA-1 in HSVECs under ALS (Figure 6A). We used EGF (100 ng/ml) to activate p-FAK expression in HSVECs; the morphology of EGF-treated HSVECs showed alignment with the ALS direction, and the peel-off of HUVECs was reduced after ALS stimulation over 24 h (Figure 6B). Western blotting showed that HSVECs upon EGF treatment could activate p-FAK and cause sustained increases in H3K9me3 expression after being subjected to ALS (Figure 6C). In addition, H3K9me3 activation was demonstrated by transfecting the H3K9me3 FRET biosensor into HSVECs under ALS. The normalized H3K9me3 FRET ratio of HSVECs was increased in the EGF group compared with that in the vehicle group after ALS stimulation. These data indicated that an increase in p-FAK activity by EGF treatment could increase H3K9me3 expression in vECs in response to the arterial flow stimuli under ALS (Figure 6D). Immunofluorescent staining of actin using phalloidin revealed a decrease in F-actin expression in vECs following ALS treatment. The supplementation of EGF rescued the effect of ALS treatment on F-actin expression and led to the formation of aligned stress fibers in parallel to the flow direction (Figure 6E). Taken together, these data indicate the involvement of FAK in H3K9me3 mediated ALS-induced vEC inflammation.

## Discussion

**FIGURE 7:**
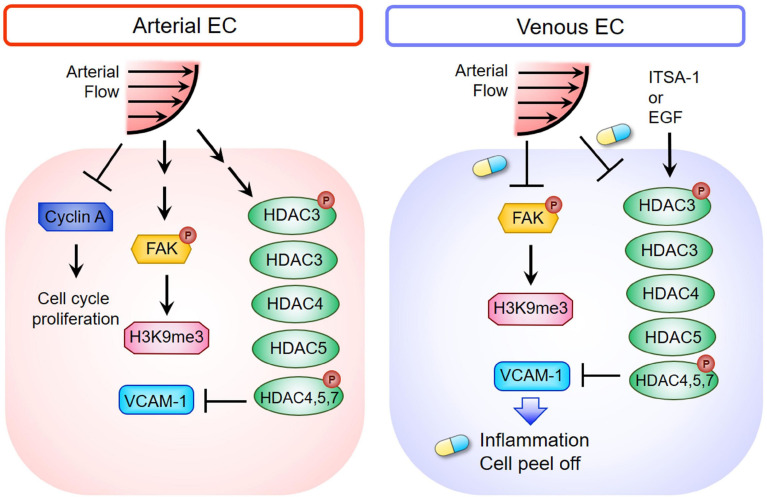
Schematic diagram depicting the mechanism of HDACs and H3K9me3-mediated inflammation in triggering vEC damage under ALS stimulation. The blue and yellow capsule symbol means that the ALS-induced damages were rescued by treating with ITSA-1 or EGF.

In the current study, we demonstrated that ALS inhibited the expressions of cyclin A1 and cyclin B1 to keep the ECs in the G0 phase, but OS did not. The responses of arterial and venous ECs to ALS stimulation were very different. Expressions of HDACs and H3K9me3 decreased in ALS-treated vECs, whereas expressions of these proteins increased in ALS-treated arterial ECs (Figure 7). It has been shown that steady laminar flow reduces EC proliferation, with cells arrested in the G0/G1 phase, whereas disturbed flow patterns increase EC turnover (Guo et al., 2007). Laminar shear stress activates the PI3K-Akt pathway and the antiproliferative adenosine monophosphate-activated protein kinase (AMPK), but in oscillatory flow, only Akt activation is found. Several studies have indicated that AMPK counteracts Akt to mediate the downstream signal of mammalian target of rapamycin (mTOR)-p70S6 kinase (S6K), which is important for the regulation of the EC cell cycle. In addition, laminar shear stress leads arterial ECs to align in the direction of flow and exhibit low turnover or activation (Givens and Tzima, 2016). The transcription factor Krüppel-like factor-2 (KLF-2), an anti-inflammatory gene, shows high expression in arterial ECs under laminar shear stress (Givens and Tzima, 2016). In contrast, disturbed flow induces activation of nuclear factor-kappa B (NFκB), VCAM-1 expression, reactive oxygen species (ROS) production, and inflammatory responses in HUVEC (Go et al., 2014). Increases of senescence-associated β-galactosidase activity and p53 expression have been documented after exposure of arterial EC to disturbed flow. These results indicate that disturbed flow upregulates the p53-p21-dependent pathway to promote senescence or apoptosis of ECs (Warboys et al., 2014). We found that laminar shear stress induced arterial ECs alignment parallel to the flow direction and reduced cell inflammation. In contrast, vECs became round and inflamed after ALS exposure.

Recent studies have demonstrated that the effects of laminar shear stress on histone modification provide the molecular basis for laminar shear stress-mediated gene regulation in ECs (Illi et al., 2003). Laminar shear stress (12 dynes/cm2) induces the phosphorylation of HDAC5 and the disassociation of p-HDAC5 and myocyte enhancer factor-2 (MEF2) in HUVECs (Wang and Wang, 2009). Upregulation of MEF2 transcriptional activity leads to the expression of KLF-2 and endothelial nitric oxide synthase (eNOS) to maintain EC functions. These studies suggested that H3K9me3 and HDACs are important factors that regulate EC function. Arterial ECs had higher expression of HDACs than vECs after ALS stimulation. Disturbed flow in arterial ECs leads to different proatherogenic phenotypes, inflammation, proliferation, apoptosis, and reduction of vascular reactivity of ECs (Heo et al., 2015). PTM and epigenetic events are involved in disturbed flow-induced endothelial proatherogenic phenotypes. Disturbed flow mediates several specific-signaling events including kinase activation (such as activation of p90ROS-sensitive kinases and inflammasome) (Heo et al., 2015), SUMOylation-related enzyme activity (Heo et al., 2013), DNA methylation (Jiang et al., 2014), and histone modification (Illi et al., 2003). Studies have indicated that laminar shear stress (10 dynes/cm^2^) promotes phosphorylation of H3S10 and acetylation of H3K14 by the formation of the cAMP-responsive element-binding protein (Illi et al., 2003; Beldjoud et al., 2015). Histone methylation is also a crucial PTM that affects a wide variety of biological processes (Heo et al., 2016). However, the specific site of histone methylation in disturbed flow has not been sufficiently investigated. We found that arterial ECs in ALS induced H3K9me3 expression, but OS and ST did not. Moreover, the increase in VCAM-1 expression was associated with a decrease in H3K9me3 expression in vECs under ALS. These results indicate that H3K9me3 expression is important in the vEC damage during transition from venous to arterial flow. H3K9me3 and HDACs may serve as critical mechanosensitive molecules to modulate inflammation and EC peel-off during transition from venous to arterial flow in vECs.

It has been shown that there is a correlation between H3K9me3 expression and tumor progression in multiple cancer types (e.g., colorectal cancer) and that global H3K9 methylation upregulates tumorigenesis through the enzymatic activity of SUV39h1 (Yokoyama et al., 2013). Dysregulation of H3K9 methylation is found in various diseases such as neurodegenerative diseases. In hepatocellular carcinoma cells, knockdown of SUV39h1 expression has been shown to decrease H3K9me3 expression and disturb cell proliferation and sphere formation (Chiba et al., 2015). Villeneuve and coworkers demonstrated that H3K9me3 and SUV39h1 have pivotal roles in repressing the expression of inflammatory genes in vascular smooth muscle cells (Villeneuve et al., 2008). It has been reported that the increase of inflammatory genes in vascular cells cultured with high glucose was accompanied with the decrease of H3K9me3, which is known to protect against the biochemical state of diabetic inflammation (Abi Khalil, 2014). In addition, studies have shown that MeCP2 suppresses cell proliferation via recruiting HDAC activity and the subsequent activation of H3K9me3 (Fuks et al., 2003). These results are consistent with our observations, which demonstrate that the upregulation of H3K9me3 can repress the inflammation and proliferation of cell, and that H3K9me3 and HDACs could reverse ALS-induced vein graft pathogenesis.

It has been revealed that short hairpin RNA-mediated knockdown of HDAC3 suppresses H3K9 methylation (Huang et al., 2011). In Hdac3-null mice, the loss of H3K9me3 was found to coincide with the failure to maintain chromatin structure (Bhaskara et al., 2010). In investigating the relationship between H3K9me3 and inflammation when ECs were subjected to ALS, we discovered that peel-off and a decrease in H3K9me3 expression occurred in vECs after subjecting them to arterial flow. Surprisingly, we observed that vECs had low expression of HDAC3 in the arterial flow-induced inflammatory response. Next, we applied ITSA-1 to vECs to induce HDAC expression. We found that the high H3K9me3 expression in vECs reduced the inflammation responses in vECs. ITSA-1 (a small molecule for HDAC activator) has been used for screening chemical genetic suppressors and could become a valuable probe of many biological processes (Koeller et al., 2003). ITSA-1 has not been applied widely in clinical studies, but it could be used to uncover new therapeutical approaches to vascular diseases. We found that H3K9me3 and HDAC could participate in the arterial flow-induced inflammatory response in vECs, but the exact mechanism is not completely understood. Further studies are needed to reveal how H3K9me3 and HDAC3 regulate ALS-induced vEC damage.

Epidermal growth factor (which is part of a complex network of growth factors and receptors) facilitates cell growth (Goodsell, 2003). In our study, EGF upregulated H3K9 methylation in vECs after ALS stimulation. Several studies have shown that EGF interacts with the epidermal growth factor receptor (EGFR) to induce its kinase activity and autophosphorylation on tyrosine residues (Ge et al., 2002). However, overexpression of EGFR engenders constitutive activation of the EGFR and has been shown to correlate with tumor proliferation, tumor metastasis, and resistance to chemotherapy (Mendelsohn and Baselga, 2000). In this regard, the clinical application of EGF treatment may be a possible approach to reduce the excessive activity of EGFR in inducing tumor proliferation.

We have provided evidence that losses of HDACs and H3K9me3 expressions might cause vEC damage under the transition of venous flow to arterial flow. Inhibition of HDAC activities in arterial ECs by a specific inhibitor caused ALS-induced inflammation and cell loss similar to that observed in vECs. Activation of HDACs and H3K9me3 by ITSA-1 treatment could prevent ALS-induced vEC peel-off and reduce VCAM-1 expression in vECs. The abnormal mechanoresponses of H3K9me3 and HDAC in vECs after ALS exposure can be reversed by ITSA-1 treatment, which could be a strategy to prevent vascular graft failure.

## Data Availability Statement

The raw data supporting the conclusions of this article will be made available by the authors, without undue reservation.

## Author Contributions

C-CW and SC conceived and supervised this project. T-YW and M-MC contributed equally to this work. T-YW, M-MC, and T-CH performed the experiments and interpreted data. T-YW, M-MC, and C-CW wrote the manuscript. SC and Y-SJL helped with data analysis and the manuscript editing. M-MC and T-CH revised the manuscript. All authors contributed to the article and approved the submitted version.

## Conflict of Interest

The authors declare that the research was conducted in the absence of any commercial or financial relationships that could be construed as a potential conflict of interest.

## Footnotes

1 www.mathworks.com/
